# PLLA/Simvastatin-Loaded Mesoporous Bioactive Glass Nanofibrous Scaffolds with Improved Osteogenic Activity and Mechanical Properties for Bone Tissue Engineering

**DOI:** 10.3390/polym18030398

**Published:** 2026-02-03

**Authors:** Wanqing Zhan, Qiqi Wen, Haiyan Yao, Junchao Wei

**Affiliations:** 1 School of Stomatology, Jiangxi Medical College, Nanchang University, Nanchang 330006, China; zhanwanqing2021@163.com (W.Z.); wqq2021max@163.com (Q.W.); ncuyaohaiyan@163.com (H.Y.); 2 Jiangxi Provincial Clinical Research Center for Oral Disease, Nanchang 330006, China; 3 Jiangxi Provincial Key Laboratory of Oral Diseases, Nanchang 330006, China; 4 School of Chemistry and Chemical Engineering, Nanchang University, Nanchang 330031, China

**Keywords:** bone tissue engineering, electrospun, PLLA, mesoporous bioactive glass

## Abstract

Insufficient osteogenic activity and mechanical properties of poly-L-lactic acid (PLLA) are urgent problems to be solved in deepening their application in bone tissue engineering. In this work, PLLA/mesoporous bioactive glass (PLLA/MBG) scaffolds and PLLA/simvastatin-loaded mesoporous bioactive glass (PLLA/MBG@SIM) scaffolds with filler content of 5, 10, and 15 wt% MBG and MBG@SIM were fabricated via electrospinning technology. At 10 wt% MBG loading, the tensile strength and tensile modulus were 3.23 ± 0.26 MPa and 124.47 ± 8.68 MPa, respectively, over 50% higher than those of PLLA scaffolds, demonstrating a significant enhancement in mechanical properties. Moreover, the incorporation of MBG improved the bioactivity of the PLLA scaffold, promoting the formation of apatite on the surface of the scaffolds. All composite scaffolds were non-toxic with good biocompatibility. Furthermore, PLLA/MBG@SIM composite scaffolds displayed superior osteogenic effects, better than the pure PLLA scaffolds and PLLA/MBG scaffolds. This work presents a multifunctional scaffold system combining enhanced mechanical strength with potent osteogenic activity, showing great promise for bone tissue engineering applications.

## 1. Introduction

Bone tissue, characterized by rich vascularization and intricate microstructure, possesses exceptional self-healing capacity, typically regenerating damaged regions while restoring original biomechanical integrity [1,2]. However, the regeneration of large bone defects remains a significant clinical hurdle, and current therapeutic strategies frequently rely on bone grafts [3]. As the gold standard for bone defects, autologous bone grafts are severely constrained by their limited supply and donor site morbidity. Allogeneic bone grafts carry risks of immune rejection and disease transmission, hindering their widespread application [4]. In recent years, bone tissue engineering (BTE) has gained much attention as a highly promising approach, and the biomaterial used for BTE scaffolds stands as a critical factor, which should possess good biocompatibility, biodegradability, inherent osteogenic potential, and proper mechanical strength [5].

Biodegradable polymers, which act as functional scaffolds, have been widely used in BTE [6]. Among them, poly-L-lactic acid (PLLA), with excellent biocompatibility and tunable biodegradability, has been widespread applied in biomedical devices, tissue engineering, and drug delivery systems [7,8]. When used in BTE, PLLA undergoes gradual degradation [9]. However, the limited osteogenic activity and inadequate mechanical performance of PLLA significantly impede its clinical translation for bone regeneration [10,11]. A prevailing strategy to overcome these limitations involves the fabrication of PLLA-based composite biomaterials, which combine the advantages of individual components [12,13]. For example, PLLA composites incorporating graphene oxide (GO), nanocrystalline hydroxyapatite (nano-HAP), and nickel oxide (NiO) nanoparticles presented superior mechanical properties and osteogenic performance than PLLA, with degradation rates matching the natural bone healing process while preserving a steady pH environment [14]. Moreover, hydroxyapatite/PLLA/decellularized extracellular matrix biocomposites exhibited improved mechanical performance and osteoinductive activity compared to PLLA [15]. These studies demonstrate that it is an efficient method to improve the performance of PLLA in BTE via combining the advantages of PLLA and other additives.

Mesoporous bioactive glass (MBG), characterized by its nanoporous structure, large specific surface area, and excellent bioactivity, represents a particularly promising bioceramic for bone regeneration [16,17,18]. Importantly, MBG can release bioactive components (Si, Ca, and PO_4_^3−^ ions), which actively modulate cellular behavior including bone cell proliferation and differentiation [19,20], and facilitate the deposition of bone-like apatite in simulated physiological fluids such as simulated body fluid (SBF) [21]. As a highly promising nanofiller, MBG can enhance osteogenic activity and significantly improve the mechanical properties of polymer matrices [22,23]. Furthermore, the porous architecture allows MBG to effectively load drugs and then release them slowly [24,25]. Simvastatin (SIM), a cholesterol-lowering drug, with potential osteoinductive effects [26,27], has been incorporated into diverse material systems to promote bone repair [28,29,30]. For example, polyvinyl alcohol scaffolds embedded with chitosan nanoparticles loaded with SIM were prepared via electrospinning technology, demonstrating superior bone-forming potential [31]. These studies implied that the integration of SIM into MBG could be a promising multifunctional nanofiller, which may improve the properties of the polymer matrix.

In this work, MBG nanoparticles were prepared and subsequently loaded with SIM to obtain MBG@SIM, and then PLLA/MBG and PLLA/MBG@SIM scaffolds with filler content of 5, 10, and 15 wt% were fabricated via electrospinning technology. The composite scaffolds exhibited superior mechanical performance and enhanced biomineralization capability compared to PLLA scaffolds. Osteogenic-related experiments further revealed that the PLLA/MBG@SIM scaffolds displayed the most remarkable osteogenic properties, better than the pure PLLA and PLLA/MBG groups, providing strong support for the expanded application of PLLA-based materials in the biomedical field, particularly in BTE applications.

## 2. Experimental Section

### 2.1. Materials and Reagents

PLLA (REVODE 110) was obtained from Zhejiang Hisun Biomaterials Co., Ltd. (Zhejiang, China). Chloroform, ethyl acetate (EA, 99.5%), *N*,*N*-Dimethylformamide (DMF), hexadecyl trimethyl ammonium bromide (CTAB, 98%), tetraethyl orthosilicate (TEOS), triethyl phosphate, calcium nitrate tetrahydrate (Ca(NO_3_)_2_·4H_2_O), ammonium hydroxide (NH_3_·H_2_O), and ethanol were purchased from Sinopharm Chemical Reagent Co., Ltd. (Shanghai, China). Simvastatin (>97%) was purchased from Aladdin Scientific Co., Ltd. (Shanghai, China).

Acridine orange/ethidium bromide (AO/EB) was purchased from BestBio Co., Ltd. (Shanghai, China). Additionally, 4% paraformaldehyde, 0.1% Triton X-100, and phosphate-buffered solution (PBS) were purchased from Solarbio Co., Ltd. (Beijing, China). TRITC-labeled phalloidin and DAPI (2-(4-amidinophenyl)-6-indolecarbamidine dihydrochloride) were purchased from Shanghai Maokang Biotechnology Co., Ltd. (Shanghai, China). Cell Counting Kit-8 (CCK-8) and ALP-Staining Kit were purchased from Beyotime Co., Ltd. (Shanghai, China).

### 2.2. Preparation of MBG and MBG@SIM

MBG synthesis was carried out following an established protocol [18]. Briefly, 0.7 g of CTAB was dissolved in 33 mL of deionized water. Subsequently, 10 mL of ethyl acetate and 7 mL of aqueous ammonia (3 mol/L) were added and stirred for 15 min. Then, 3.6 mL of TEOS, 0.36 mL of triethyl phosphate, and 2.277 g of (Ca(NO_3_)_2_·4H_2_O) were added to the mixture sequentially at 30 min intervals. The resulting mixture was stirred for 4 h, and then the precipitate was collected by centrifugation, washed three times with ethanol and deionized water, and finally dried at 60 °C. The dried powder was finally calcined at 650 °C for 6 h to yield MBG. To obtain MBG@SIM, 10 mg of MBG was ultrasonically dispersed in 8 mL of anhydrous ethanol, while 2 mg of SIM was dissolved in 2 mL of anhydrous ethanol. After mixing, the solution was magnetically stirred for 24 h, and then the precipitate was collected via centrifugation and lyophilization.

### 2.3. Characterization of MBG and MBG@SIM

The morphology of nanoparticles was observed by scanning electron microscopy (SEM, Thermo Fisher Scientific, Apero C HiVac, Waltham, MA, USA). A high-vacuum physisorption analyzer (Quantachrome, autosorb IQ203030846, Boynton Beach, FL, USA) according to Brunauer–Emmett–Teller (BET) was employed to determine the specific surface area, pore size distribution, and the pore volume.

### 2.4. Fabrication of Fibrous Scaffolds

Firstly, 1.2 g of PLLA was dissolved in 8 mL of chloroform and the mixture was stirred for 24 h, while MBG and MBG@SIM was dispersed in 2 mL DMF, respectively. Then, each dispersion was mixed with 8 mL of PLLA solution and homogenized by gentle stirring, with the content of MBG and MBG@SIM being 5, 10, and 15 wt%, and the composites were denoted as 5 MBG/PLLA, 10 MBG/PLLA, 15 MBG/PLLA, 5 MBG@SIM/PLLA, 10 MBG@SIM/PLLA, and 15 MBG@SIM/PLLA, respectively. The solution was added into a syringe and placed on the syringe pump for electrospinning. The voltage was set at 18 kV, and the flow rate was adjusted to 1 mL/h. Finally, the fibrous scaffolds were dried under vacuum at 37 °C.

### 2.5. Characterization of Fibrous Scaffolds

The surface morphology analysis was performed on all scaffolds by SEM. Fiber diameter was determined by analyzing 100 randomly selected fibers from the micrographs. PLLA/MBG scaffolds were further characterized by Fourier-transform infrared spectroscopy (FTIR), water contact angle (WCA) testing, mechanical testing, and thermogravimetric analysis (TGA). FTIR spectra were obtained via a Fourier transform infrared spectrometer (JASCO, FT/IR-4700, Hachioji, Tokyo, Japan). The surface wettability was evaluated by a contact angle measuring instrument (KINO, SL200KS, New York, NY, USA). Mechanical properties were evaluated using a universal testing machine (Instron, CH17-621, Norwood, MA, USA). For each group, five 1 cm × 5 cm specimens were evaluated at 2 mm/min in dry air (25 °C, 40% RH). The thermal stability was measured by thermal gravimetric analyzer (TA Instruments, Discovery TGA55, New Castle, DE, USA) under a nitrogen atmosphere with samples that were heated from 0 to 180 °C at a heating rate of 10 °C/min.

### 2.6. Biomineralization Evaluation

Square specimens (1 × 1 cm^2^) were cut from the scaffolds and immersed in 10 mL of 1.5 × simulated body fluid (1.5 × SBF) for 7 days, with daily solution renewal. After incubation, the samples were rinsed with deionized water, vacuum-dried, and examined by SEM for apatite formation on the surface.

### 2.7. Culture of Cells

The MC3T3-E1 cell line was obtained from Shanghai Fuheng Biotechnology Co., Ltd. (Shanghai, China) and cultured in α-MEM supplemented with 10% FBS at 37 °C under 5% CO_2_.

### 2.8. Biocompatibility Testing

Cell viability was evaluated via the CCK-8 assay and acridine orange/ethidium bromide (AO/EB) live/dead staining. For the CCK-8 assay, fibrous scaffolds were punched into 6 mm diameter disks, sterilized under ultraviolet light for 1 h, and rinsed with PBS. At a density of 4 × 10^4^ cells/well, MC3T3-E1 cells were plated onto the scaffolds in 96-well plates, and the wells without scaffolds served as the controls. After 48 h of incubation, the culture medium was replaced with CCK-8 working solution, and the plates were further incubated for 2 h. The absorbance was then measured at 450 nm using a microplate reader. For AO/EB staining, MC3T3-E1 cells were seeded at a higher density of 8 × 10^4^ cells/well on sterile scaffolds. After 48 h of culture, the cells were stained with AO/EB solution, washed with PBS, and observed under a fluorescence microscope (Leica, DMi8, Wetzlar, Germany).

Cell adhesion behaviors were evaluated by cytoskeleton staining. MC3T3-E1 cells were seeded on the scaffolds at 8 × 10^4^ cells/well and cultured for 48 h. Subsequently, the samples were washed with PBS, and the cells were fixed with 4% paraformaldehyde for 10 min. Then, the cells were permeabilized with 0.1% Triton X-100 for 5 min. F-actin and nuclei were respectively stained with TRITC-labeled phalloidin (20 min) and DAPI (3 min). Cell morphology and adhesion were then observed by fluorescence microscopy.

### 2.9. Osteogenic Properties

#### 2.9.1. Alkaline Phosphatase (ALP) Activity Assay

After 7 days of osteogenic induction, the MC3T3-E1 cells cultured on the samples were fixed with 4% PFA. ALP staining was then performed using a BCIP/NBT alkaline phosphatase color development kit (Beyotime, Shanghai, China). The samples were washed twice with deionized water and dried, and then the images were taken on a stereoscopic microscope (Leica, EZ4W, Wetzlar, Germany).

#### 2.9.2. Expression of Osteogenesis-Related Genes

Quantitative real-time polymerase chain reaction (qRT-PCR) was used to assess the expression of osteogenesis-related genes in MC3T3-E1 cells on nanofiber scaffolds, including bone morphogenetic protein 2 (*Bmp-2*), Runt-associated transcription factor 2 (*Runx2*), and osteocalcin (*Ocn*). Briefly, MC3T3-E1 cells (1 × 10^4^ cells/mL) were seeded on scaffolds and cultured for 7 days. Total RNA was extracted using TRIZOL (Beyotime, Shanghai, China), reverse-transcribed with a PrimeScript RT kit (Vazyme Biotech, Nanjing, China), and amplified via SYBR Premix Ex *Taq*II kit (Vazyme Biotech, Nanjing, China). Primer sequences are listed in Table 1. Relative expression was calculated via the 2^−ΔΔCt^ method.

### 2.10. Statistical Analysis

Data are presented as mean ± standard deviation. Comparisons between two groups were performed via Student’s *t*-test, while differences among three or more groups were assessed via one-way ANOVA followed by Tukey’s post hoc test. A *p*-value < 0.05 was considered statistically significant, with greater significance denoted by ** *p* < 0.01 and *** *p* < 0.001.

## 3. Result and Discussion

### 3.1. Characterization of MBG and MBG@SIM

**Figure 1:**
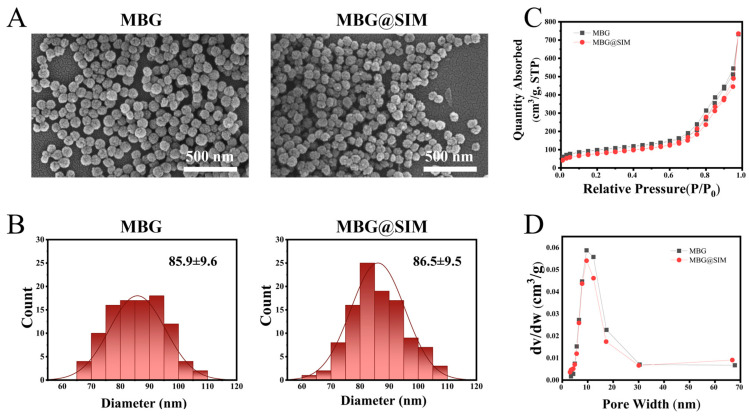
Characterizations of MBG and MBG@SIM. (**A**) SEM images (scale bar represents 500 nm), (**B**) particle size distribution, (**C**) N_2_ adsorption–desorption isotherms, and (**D**) pore size distribution curves of MBG and MBG@SIM.

SEM images showed both MBG and MBG@SIM were spherical particles with good dispersion and uniform size, showing average diameter of 85.9 ± 9.6 nm and 86.5 ± 9.5 nm, respectively (Figure 1A,B). Both MBG and MBG@SIM displayed distinctive Type IV isotherms with H3-type hysteresis loops, confirming their mesoporous structure (Figure 1C). The pore size distribution showed average diameters of 15.49 nm for MBG and 16.42 nm for MBG@SIM (Figure 1D, Table 2). The specific surface area of MBG and MBG@SIM were 285.68 m^2^/g and 276.99 m^2^/g, respectively, while the pore volume of MBG and MBG@SIM were 1.11 cm^3^/g and 1.14 cm^3^/g, indicating that low SIM loading showed negligible influence on the mesopore architecture of MBG.

### 3.2. Morphology of Fibrous Scaffolds

**Figure 2:**
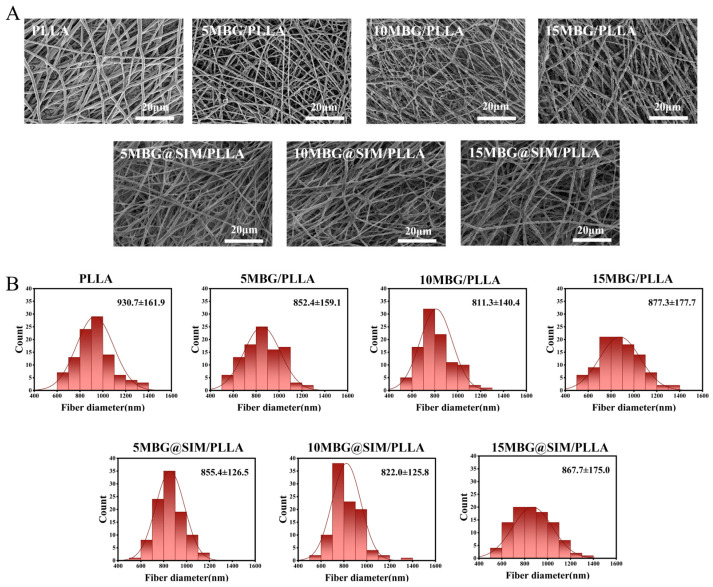
Morphology of fibrous scaffolds. (**A**) SEM images and (**B**) fiber diameter distribution of PLLA, 5 MBG/PLLA, 10 MBG/PLLA, 15 MBG/PLLA, 5 MBG@SIM/PLLA, 10 MBG@SIM/PLLA, and 15 MBG@SIM/PLLA. Scale bar represents 20 µm.

All scaffolds showed randomly arranged nanofibers forming 3D porous structures (Figure 2A). The EDS mapping of PLLA scaffolds exhibited only carbon and oxygen signals, whereas the MBG-incorporated PLLA scaffolds displayed characteristic signals of silicon, calcium, and phosphorus, confirming the successful incorporation of MBG within the PLLA fibers (Figure S1). The fiber diameter of PLLA was 930.7 ± 161.9 nm (Figure 2B). As MBG or MBG@SIM loading increased, the fiber diameters became 852.4 ± 159.1 nm for 5 MBG/PLLA, 855.4 ± 126.5 nm for 5 MBG@SIM/PLLA, 811.3 ± 140.4 nm for 10 MBG/PLLA, 822.0 ± 125.8 nm for 10 MBG@SIM/PLLA, 877.3 ± 177.7 nm for 15 MBG/PLLA, and 867.7 ± 175.0 nm for 15 MBG@SIM/PLLA. The minimum diameter occurred at 10 wt% filler loading, suggesting that moderate filler content may optimize solution properties for finer fiber formation, while higher loadings (15 wt%) led to a slight increase of fiber diameter, which could be due to altered solution homogeneity. Overall, MBG and MBG@SIM exhibited similar effects on fiber morphology, with SIM loading showing negligible influence on diameter. Notably, as MBG or MBG@SIM content increased, fiber surfaces became progressively rougher. Substrates with a certain degree of surface roughness were found to promote the adhesion of osteoblasts, which could enhance the interfacial adhesion between cells and the scaffold matrix [32]. Moreover, these nanofibrous scaffolds exhibited interconnected microfiber networks offering large surface-area-to-volume ratios, which mimicked physical architecture of native extracellular matrix (ECM), supporting cell adhesion and proliferation as well as guiding tissue formation [33].

### 3.3. Physicochemical Properties

**Figure 3:**
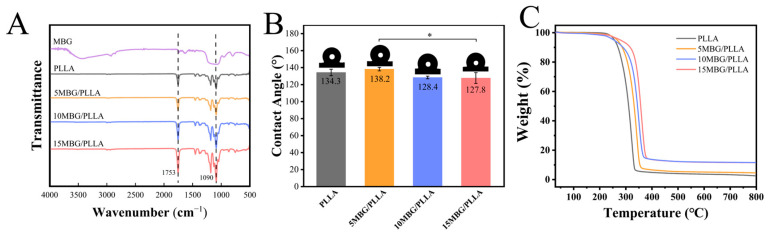
(**A**) FTIR spectra of MBG, PLLA, 5 MBG/PLLA, 10 MBG/PLLA, and 15 MBG/PLLA. (**B**) Water contact angle (n = 3), (**C**) TGA curves of PLLA, 5 MBG/PLLA, 10 MBG/PLLA, and 15 MBG/PLLA. Data are presented as mean ± SD. Statistical significance was analyzed using one-way ANOVA (* *p* < 0.05).

The FT-IR spectrum of pure PLLA exhibited a characteristic absorption band at 1753 cm^−1^ (Figure 3C), corresponding to the C=O stretching vibration of its ester groups. Meanwhile, a characteristic peak at 1090 cm^−1^ was observed in MBG and MBG-containing scaffolds, which was attributed to Si-O-Si asymmetric stretching vibrations. Furthermore, the absorption peak became stronger progressively with higher MBG content, indicating homogeneous MBG distribution in the composites.

The surface wettability of scaffolds plays a critical role in mediating cell affinity [34]. The water contact angle of all scaffolds was more than 90°, indicating that the surfaces were hydrophobic (Figure 3B). Specifically, the water contact angle of PLLA was 134.3 ± 3.8°. Upon the addition of 5 wt% MBG, the water contact angle became 138.2 ± 2.1°. When the MBG content increased to 10 and 15 wt%, the water contact angle decreased to 128.4 ± 1.6° and 127.8 ± 6.3°, respectively. The water contact angle of the scaffold can be influenced by its material composition and surface morphology [35]. The trend of the water contact angle first increasing and then decreasing can be attributed to the effects of increased surface roughness resulted from MBG, which can amplify intrinsic hydrophobicity, and the inherently hydrophilic nature of the bioceramic itself [36,37]. When the MBG content was low, the hydrophobic effect caused by the increased roughness became dominant. Instead, when the MBG content was high, the hydrophilic effect brought by the material composition dominated.

The thermal stability of composite scaffolds was studied by thermogravimetric analysis (TGA). TGA curves of PLLA, 5 MBG/PLLA, 10 MBG/PLLA, and 15 MBG/PLLA are shown in Figure 3C. The temperatures at 5% weight loss are summarized in Table 3. Specifically, the decomposition temperatures at 5% weight loss of PLLA was 256.5 °C. Upon the addition of 5 wt% MBG, the decomposition temperatures became 267.0 °C. With MBG content at 15 wt%, the decomposing temperature reached 279.2 °C, representing an increase of 22.7 °C compared to that of pure PLLA. These results revealed that the incorporation of MBG could enhance the thermal stability of PLLA scaffolds, which may affect the processing technology or conditions and mechanical properties.

**Figure 4:**
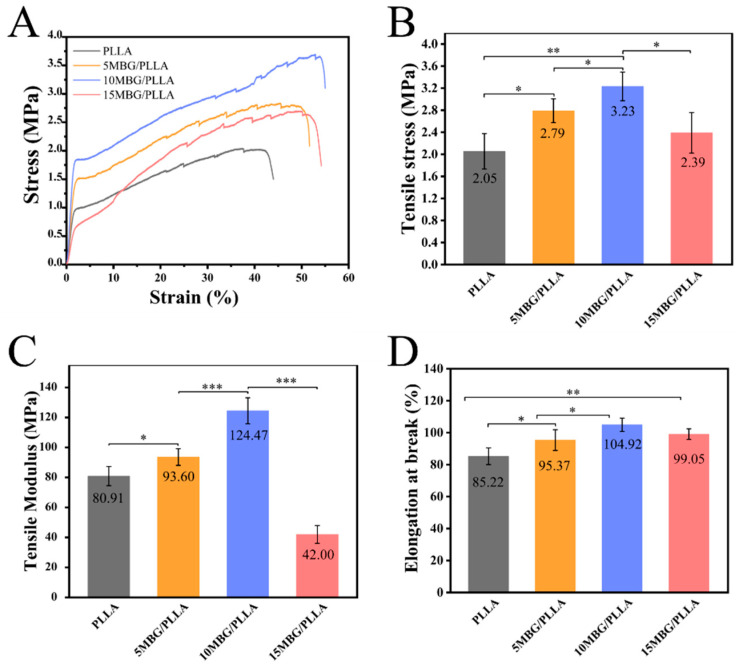
Mechanical properties of composite scaffolds. (**A**) Stress–strain curves, (**B**) tensile strength, (**C**) tensile modulus, and (**D**) elongation at break of PLLA, 5 MBG/PLLA, 10 MBG/PLLA, and 15 MBG/PLLA (n = 5). Data are presented as mean ± SD. Statistical significance was analyzed using one-way ANOVA. (* *p* < 0.05, ** *p* < 0.01, *** *p* < 0.001).

The influences of MBG on the tensile properties of PLLA were evaluated through a tensile test. The stress–strain curve, tensile stress, tensile modulus, and elongation at break of the composite scaffolds are presented in Figure 4. The tensile strength of pure PLLA was 2.05 ± 0.32 MPa (Figure 4B). Upon MBG addition, the tensile strength first increased, reaching an optimal value of 3.23 ± 0.26 Mpa at 10 wt% loading, exhibiting 57.56% higher than pure PLLA. However, at a higher loading of 15 wt%, the strength decreased to 2.39 ± 0.37 Mpa. Similarly, the tensile modulus was 124.47 ± 8.68 Mpa when the MBG content reached 10 wt% (Figure 4C), representing 53.84% higher than pure PLLA (80.91 ± 6.39 Mpa). All MBG-containing scaffolds exhibited higher elongation at break than pure PLLA, with the maximum value of 104.92 ± 4.19% achieved at 10 wt% MBG loading (Figure 4D), indicating that an appropriate amount of MBG can enhance the toughness of PLLA. The results of the mechanical tests demonstrated MBG could enhance the mechanical properties of PLLA scaffolds, and the enhanced efficiency was found to be related to the MBG contents. As the content of MBG increased from 0 wt% to 15 wt%, the mechanical properties first increased and then decreased, with the optimum mechanical properties occurring at 10 wt%. The enhanced mechanical properties at 10 wt% MBG can be attributed to effective transfer of stress from the PLLA matrix to well-dispersed MBG particles acting as reinforcing fillers. With MBG content reaching 15 wt%, the decline of mechanical properties could relate to SEM observations in which 15 wt% MBG incorporation induced excessive fiber roughening, nanoparticle agglomeration, and heterogeneous MBG distribution, collectively compromising structural integrity (Figure 2). These defects likely initiated premature failure under tensile loading, as evidenced by the reduced elongation at break (Figure 4D). As a matter of fact, it is a common phenomenon that the nanoparticles would aggregate in the polymer matrix [38]. To conquer this problem, great efforts have been made to modify the surface of nanofillers so that they can disperse well in the polymer matrix, which may improve the mechanical properties of polymer composites. In our prior studies, we have proposed efficient methods to tune the surface properties of graphene and SiO_2_ by grafting polymer chains on their surface, realizing the homogenous dispersion of nanofillers in PLLA matrix [39,40]. Therefore, we are sure that similar strategy applied to MBG could also be a promising direction to overcome the current dispersion limit and further improve composite performance in future work.

### 3.4. Biomineralization Evaluation

**Figure 5:**
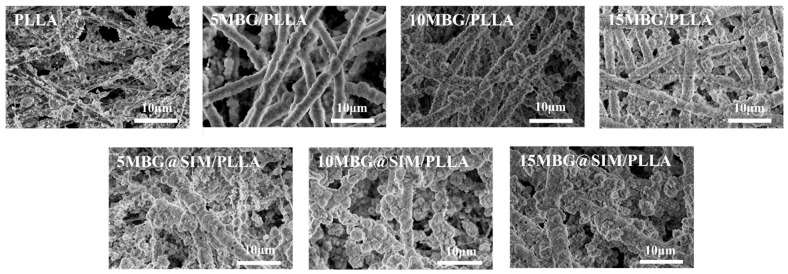
SEM images of fibrous scaffolds treated with 1.5 × SBF for 7 days. Scale bar represents 10 µm.

An important aspect of tissue engineering scaffolds for bone regeneration is their bioactivity, which can be assessed by their capacity to form a bone-like apatite layer in SBF [41]. Herein, after immersion in SBF at 37 °C for 7 days (with daily solution renewal), the scaffolds were dried and examined by SEM. SEM images showed sparse hydroxyapatite (HA) crystals on PLLA, whereas dense HA coatings formed on MBG-containing scaffolds (Figure 5). With MBG or MBG@SIM content reaching 15 wt%, a continuous HA layer that fully encapsulated the fibers was observed. The results demonstrated that MBG enhanced the bioactivity of PLLA scaffolds in a concentration-dependent manner, with higher MBG content yielding significantly greater hydroxyapatite formation. This enhanced bioactivity can be attributed to the ions (Ca^2+^ and PO_4_^3−^) released by MBG, which initiated HA nucleation from the solution [19].

### 3.5. In Vitro Biocompatibility

**Figure 6:**
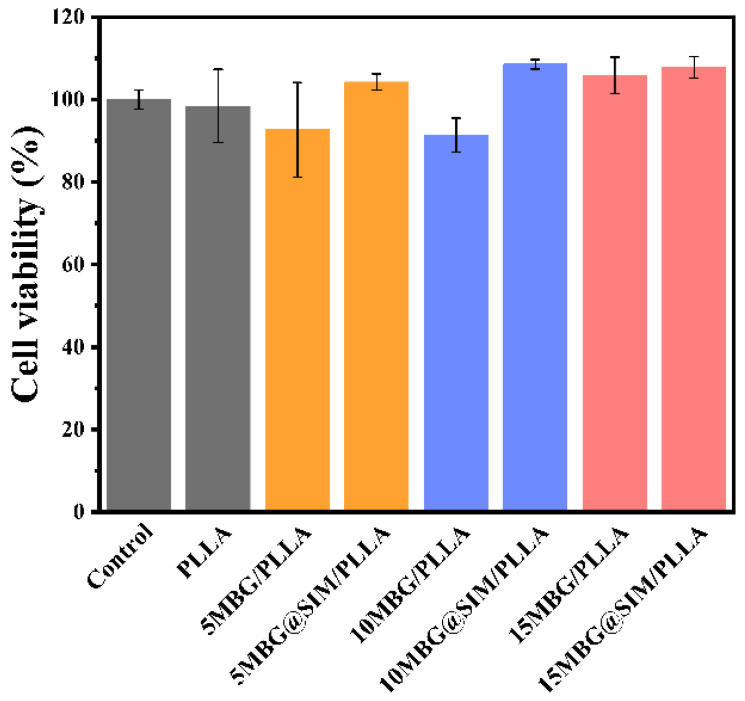
Cell viability results of MC3T3-E1 cells on different samples (n = 3). Data are presented as mean ± SD. Statistical significance was analyzed using one-way ANOVA.

**Figure 7:**
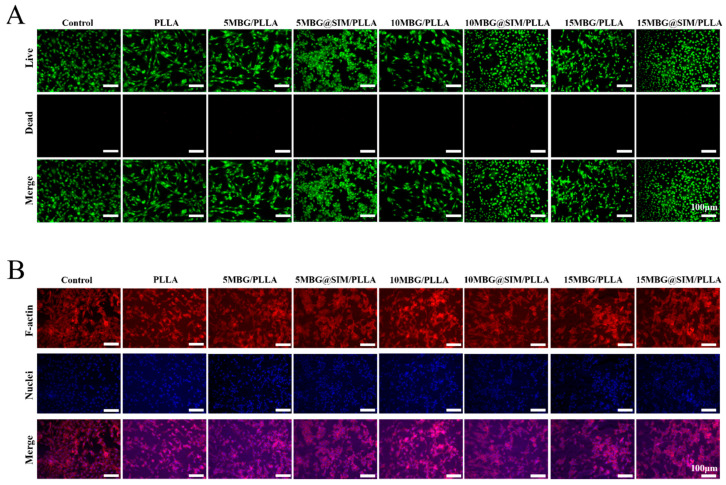
(**A**) AO/EB staining and (**B**) cell adhesion results of MC3T3-E1 cells on different samples. Scale bar represents 100 µm.

To ensure the inherent biocompatibility required for bone scaffolds, we evaluated the biocompatibility of the composite scaffolds using a CCK-8 cell viability assay, AO/EB live-dead staining, and a cell adhesion test on MC3T3-E1 cells. As shown in Figure 6, the cell viabilities of MC3T3-E1 cells for PLLA, 5 MBG/PLLA, 5 MBG@SIM/PLLA, 10 MBG/PLLA, 10 MBG@SIM/PLLA, 15 MBG/PLLA, and 15 MBG@SIM/PLLA were 98.4 ± 8.93%, 92.66 ± 11.45%, 104.21 ± 1.99%, 91.35 ± 4.12%, 108.53 ± 1.16%, 105.82 ± 4.46%, and 107.72 ± 2.61%, respectively. All composite scaffolds showed no significant difference from the control group, demonstrating their good biocompatibility. AO/EB live-dead staining, which labels live cells green with acridine orange (AO) and dead cells red with ethidium bromide (EB), further supported the favorable biocompatibility of all scaffolds, with minimal red fluorescence observed across them, demonstrating high cell viability (Figure 7A). As for the cell adhesion test, cytoskeleton staining was employed to assess cell adhesion behavior across all groups. Compared with the groups of control and PLLA, MC3T3-E1 cells on other MBG-containing scaffolds exhibited elongated, spread morphologies with distinct F-actin cytoskeletons (Figure 7B), demonstrating that MBG incorporation could enhance cell–material interactions. Our findings confirmed that composite scaffolds can promote cell adhesion while maintaining favorable biocompatibility and non-cytotoxicity toward MC3T3-E1 cells. These results validate their translational promise for bone regeneration applications.

### 3.6. In Vitro Osteogenic Differentiation

**Figure 8:**
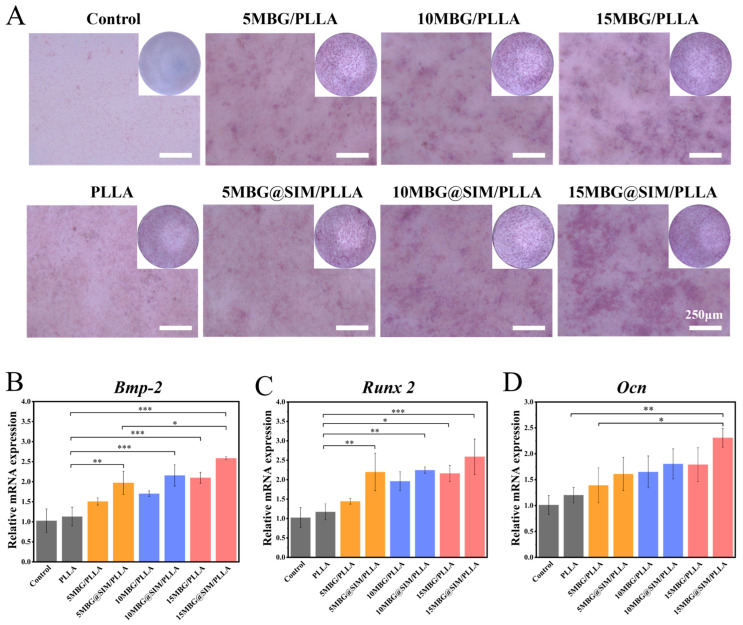
The osteogenic properties of fibrous scaffolds. (**A**) ALP staining of MC3T3-E1 cells of different groups after 7 days of culture. (**B**–**D**) Relative gene expressions levels of *Bmp-2*, *Runx2*, and *Ocn* in MC3T3-E1 cells cultured on different samples for 7 days. (n = 3). Scale bar represents 250 µm. Data are presented as mean ± SD. Statistical significance was analyzed using one-way ANOVA. (* *p* < 0.05, ** *p* < 0.01, *** *p* < 0.001).

To assess the pro-osteogenic properties of composite scaffolds on MC3T3-E1 cells, we performed ALP staining, ALP activity quantification, and qRT-PCR analysis of osteogenic differentiation markers. As an early biomarker in osteogenesis, ALP is crucial for promoting extracellular matrix mineralization [42]. Following 7 days of osteogenic induction, minimal purple staining was observed in the control and PLLA groups, corresponding to low ALP activity (Figure 8A). The intensity of purple staining increased progressively with higher MBG and MBG@SIM content, and the MBG@SIM-incorporated scaffolds exhibited deeper purple staining than those incorporating MBG alone, indicating enhanced osteogenic activity. The qRT-PCR was employed to detect the expression of osteogenic-related genes including bone morphogenetic protein-2 (*Bmp-2*), Runt-related transcription factor 2 (*Runx2*), and osteocalcin (*Ocn*) of MC3T3-E1 cells after 7 days of osteogenic induction with different samples. The result showed that 15 MBG@SIM/PLLA group exhibited highest expression levels of *Bmp-2*, *Runx2*, and *Ocn* (Figure 8B–D). These results indicated that PLLA/MBG@SIM fibrous scaffolds can promote osteogenic differentiation of MC3T3-E1 cells, with promising potential for BTE.

## 4. Conclusions

In summary, we fabricated a series of PLLA/MBG scaffolds and PLLA/MBG@SIM scaffolds with filler content of 5, 10, and 15 wt% MBG and MBG@SIM for bone regeneration applications. MBG can reinforce the mechanical properties of the PLLA scaffolds, and the reinforcing efficiency was related to the content of MBG. Specifically, the addition of 10 wt% MBG significantly improved tensile strength and modulus by 57.56% and 53.84%, respectively, compared to PLLA scaffolds. Moreover, these composite scaffolds exhibited favorable cytocompatibility, with escalating biomineralization ability and osteogenic performance corresponding to increased bioactive filler contents. Crucially, PLLA/MBG@SIM scaffolds displayed the most pronounced osteogenic effect, better than that of both PLLA scaffolds and PLLA/MBG scaffolds. Collectively, PLLA/MBG@SIM electrospun nanofibrous scaffolds with improved mechanical strength, superior osteogenic activity, and favorable biocompatibility present significant translational potential for BTE applications.

## Figures and Tables

**Table 1:** The primers of genes used for MC3T3-E1 cells.

Gene	Forward Primer Sequence (5′-3′)	Reverse Primer Sequence (5′-3′)
*Bmp-2*	TGAGGATTAGCAGGTCTTTGC	GCTGTTTGTGTTTGGCTTGA
*Runx2*	CTCTTCCCAAAGCCAGAGTC	CAGCGTCAACACCATCATTC
*Ocn*	CCGGGAGCAGTGTGAGCTTA	TAGATGCGTTTGTAGGCGGTC
*GAPDH*	ACCCAGAAGACTGTGGATGG	CACATTGGGGGTAGGAACAC

**Table 2:** Specific surface area, pore volume, and pore size of MBG and MBG@SIM.

Sample	Specific Surface Area (m^2^ g^−1^)	Pore Volume (cm^3^ g^−1^)	Pore Size (nm)
MBG	285.68	1.11	15.49
MBG@SIM	276.99	1.14	16.42

**Table 3:** The decomposition temperatures at 5% weight loss of fibrous scaffolds.

Sample	Temperature (°C) at 5% Weight Loss
PLLA	256.5
5 MBG/PLLA	267.0
10 MBG/PLLA	254.2
15 MBG/PLLA	279.2

## Data Availability

The original contributions presented in this study are included in the article. Further inquiries can be directed to the corresponding author.

## Supplementary Materials

The following supporting information can be downloaded at: https://www.mdpi.com/article/10.3390/polym18030398/s1, Figure S1: EDS mapping of PLLA, 5 MBG/PLLA, 10 MBG/PLLA, 15 MBG/PLLA. Scale bar represents 4 µm.
