# Supplementary material for: PLLA/Simvastatin-Loaded Mesoporous Bioactive Glass Nanofibrous Scaffolds with Improved Osteogenic Activity and Mechanical Properties for Bone Tissue Engineering
Source: Polymers (Basel). 2026 Feb 3;18(3):398. doi: 10.3390/polym18030398 (PMC12899384; doi:10.3390/polym18030398)
Supplement: Supplementary file 1 [file polymers-18-00398-s001.zip › polymers-4120511-supplementary.pdf]

Supporting Information

PLLA/simvastatin-loaded mesoporous bioactive glass nanofibrous scaffolds with improved osteogenic activity and mechanical properties for bone tissue engineering

Wanqing Zhan [a,b,c #], Qiqi Wen [a,b,c,d #], Haiyan Yao [a,b,c,d], Junchao Wei [a,b,c,d*]

[a] *School of Stomatology, Jiangxi Medical College, Nanchang University, Nanchang 330006, China*

[b] *Jiangxi Provincial Clinical Research Center for Oral Disease, Nanchang 330006, China*

c Jiangxi Provincial Key Laboratory of Oral Diseases, Nanchang 330006, China

d School of Chemistry and Chemical Engineering, Nanchang University, Nanchang 330031, China

[#] *These two authors contributed equally to this work*

** Corresponding author: weijunchao@ncu.edu.cn.*

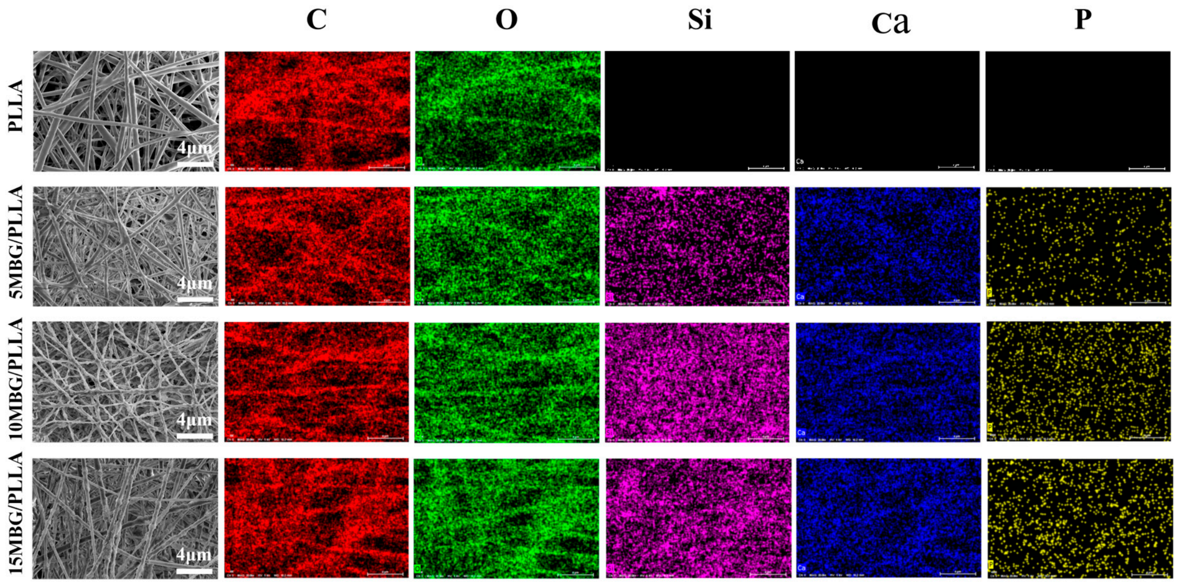

Figure S1. EDS mapping of PLLA, 5MBG/PLLA, 10MBG/PLLA, 15MBG/PLLA. Scale bar represents 4 μm.
